# The utility of sTREM-1 and presepsin to predict infection in pediatric patients receiving mechanical circulatory support

**DOI:** 10.1051/ject/2025008

**Published:** 2025-06-16

**Authors:** Robert Murray, Jianli Bi, Robin Alexander, Md Rejuan Haque, Brian Beckman, Ruth Seabrook, W. Joshua Frazier, Andrew R. Yates

**Affiliations:** 1 Nationwide Children’s Hospital 700 Children’s Drive Columbus OH 43205 USA; 2 Nationwide Children’s Hospital, Molecular Core for Cardiovascular Research 700 Children’s Drive Columbus OH 43205 USA; 3 Nationwide Children’s Hospital, Heart Center 700 Children’s Drive Columbus OH 43205 USA; 4 Biostatistics Resource at Nationwide Children’s Hospital 700 Children’s Drive Columbus OH 43205 USA; 5 Department of Biomedical Informatics, Center for Biostatistics at The Ohio State University 1800 Children’s Drive Columbus OH 43210 USA; 6 The Ohio State University College of Medicine, Department of Pediatrics, Division of Neonatology 1645 Neil Ave Columbus OH 43210 USA; 7 The Ohio State University College of Medicine, Department of Pediatrics, Section of Critical Care Medicine 1645 Neil Ave Columbus OH 43210 USA; 8 The Ohio State University College of Medicine, Department of Pediatrics, Section of Cardiology 1645 Neil Ave Columbus OH 43210 USA

**Keywords:** ECMO, Mechanical circulatory support, sTREM-1, Presepsin, Infection

## Abstract

*Background*: It is difficult to clinically detect a new infection in patients with Mechanical Circulatory Support (MCS; including veno-arterial and veno-veno extracorporeal membrane oxygenation, and ventricular assist devices). The prompt, accurate identification of new infection utilizing plasma biomarkers could prompt earlier initiation of antimicrobial agents and may improve outcomes. *Methods*: We utilized ELISA to evaluate novel biomarkers, soluble Triggering Receptor Expressed on Myeloid cells (sTREM-1) and Presepsin, as well as existing biomarkers (C-Reactive Protein (CRP) and Procalcitonin) before MCS, daily for the first week of MCS and for the 72 h in advance of the development of a new infection for patients prospectively enrolled in a biobank and who developed a culture positive infection. *Results*: Serial samples from 18 patients were analyzed. On average post-cannulation Presepsin and sTREM-1 values were not significantly different, however they have higher baseline values than reported in other patient populations. On average during periods of infection, Presepsin was 41% lower (51,462–30,188 pg/mL) (*P* = 0.001) and procalcitonin was 51% lower (0.77–0.38 ng/mL) (*P* < 0.001) compared to non-infected periods. Neither CRP or sTREM-1 were significantly different between infected and un-infected periods. *Conclusion*: Presepsin and Procalcitonin decreased in advance of the development of a new infection in the MCS patient population, a direction of change different than expected. These findings highlight the importance of biomarker studies specifically performed in the MCS patient population, and the potential lack of translatability of biomarkers in other patient populations to the MCS patient population.

**Abbreviations**
CRP: C-Reactive Protein
ECPR: cannulation to MCS during the process of active cardiopulmonary resuscitation
MCS: Mechanical Circulatory Support
sTREM-1: soluble Triggering Receptor Expressed on Myeloid cells
VAD: Ventricular Assist Device

## Introduction

Mechanical Circulatory Support (MCS), including: veno-arterial and veno-veno extracorporeal membrane oxygenation, and ventricular assist devices, is utilized to provide support for the heart, lungs or both for the most critically ill children in the intensive care unit [1]. Survival to hospital discharge in those who received MCS ranges from 41 to 74% with neonatal respiratory indications having the best outcome and ECPR (cannulation to MCS during the process of active cardiopulmonary resuscitation) having the worst [2]. The development of new infection on MCS affects up to 64% (8–64%) of patients and has been shown to impact outcomes [3, 4]. Those who develop an infection while on MCS have a longer duration of support, require longer post-MCS mechanical ventilation and have a higher mortality rate [3]. Early detection of infection and prompt initiation of antibiotics are necessary to improve outcomes in MCS patients [5, 6]. There is considerable variability between centers on how to monitor or when to start treatment for suspected infection in patients receiving MCS [7]. The Extracorporeal Life Support Organization recommends against the practice of routine blood, urine or sputum cultures and acknowledges the difficulty that MCS places on the use of traditional methods to diagnose infection, including the unknown significance of inflammatory markers.

The effectiveness of the commonly utilized biomarkers C-Reactive Protein (CRP) and Procalcitonin, in aiding with the diagnosis of infection during MCS is fraught with limitations and remains under investigation [7–11]. These biomarkers face limitations specifically due to the effect of MCS-induced inflammation on CRP and Procalcitonin kinetics [4, 7, 11, 12]. Previous pediatric studies have shown mixed results regarding the predictive value of CRP and Procalcitonin for new infection during MCS [8, 9]. One such study, Bobillo et al. did find that procalcitonin decreased after cannulation in patients with elevated pre-cannulation procalcitonin levels, while in patients with low pre-cannulation procalcitonin levels the levels remained stable; this same paper did not identify an increase in procalcitonin levels in response to infection [11].

Soluble Triggering Receptor Expressed on Myeloid Cells (sTREM-1) and Presepsin (soluble CD14 Subtype) have been demonstrated to be promising emerging biomarkers of infection, given their ability to differentiate new infection from other types of inflammatory responses [6, 13]. sTREM-1 and Presepsin are two cell surface biomarkers released during periods of innate immune cell activation, such as invasive infection, which are detectable in plasma [6, 14–17]. sTREM-1 has the potential to be a specific marker for invasive infection rather than inflammation alone due to the synergistic mechanism of sTREM-1 acting with Toll Like Receptors and aiding their recognition of foreign pathogens [15]. Presepsin is expressed and released as a result of macrophage binding to pathogenic organisms, again giving Presepsin potential to more specifically identify invasive infection rather than inflammation alone [14]. In septic neonates not on MCS, both Presepsin and sTREM-1 were significantly elevated compared to non-septic neonates and these biomarkers had greater sensitivity and specificity compared to CRP in detecting infection [6]. Presepsin was elevated in children with infection compared to those with non-infectious systemic inflammatory response syndrome [18, 19]. We aim to investigate the utility of the novel biomarkers sTREM-1 and Presepsin in detecting infection during MCS. We evaluated the kinetics of sTREM-1 and Presepsin during cannulation for MCS and compared them to current commonly used biomarkers CRP and Procalcitonin. We hypothesize that increases in sTREM-1 and Presepsin will predict infection in advance of culture proven infection in pediatric MCS patients.

## Methods

Institutional Review Board (IRB) approval with a waiver of informed consent was obtained (IRB number: STUDY00002511). Patients were included if they received MCS between 2013 and 2021 at our institution regardless of indication, had previously provided consent to participate in the MCS biomarker study (all patients receiving MCS at our institution were eligible for inclusion in this study unless they weighed less than 2 kg or were of Jehovah’s witness faith [or another faith which were against transfusions]), has included consent for the use of residual samples and had acquired a new infection during MCS. Exclusion criteria included the lack of consent for the use, or the absence of residual samples at appropriate times points. Strengthening the Reporting of Observation studies in Epidemiology (STROBE) guidelines reporting guidelines were utilized as a structured framework for reporting this project [20].

### ECMO and VAD circuit priming

We utilize the CentriMag and PediMag circuits for ECMO patients at our institution. For patients weighing less than 15 kg we utilize 2 units of Packed Red Blood Cells (PRBC), 1 unit of Fresh Frozen Plasma (FFP) and 120 mL of Platelets. For patients weighing more than 15 kg we utilize 3 units of PRBC and 1 unit of FFP. We will also utilize 25% albumin, Calcium Chloride and Sodium Bicarbonate in order to balance the circuit prime. Patients will typically be loaded with 50–100 units/kg of heparin and anticoagulation will be maintained with Bivalirudin once the activated clotting time and partial thromboplastin time is within range and the patient is not experiencing bleeding. We utilize multiple VAD circuits at our institution including: CentriMag/PediMag, Berlin Hearts, HeartMate 3 and Impella pumps. We only prime our CentriMag/PediMag with potentially 1 unit of PRBC however this is surgeon and case dependant based on the dilutional hematocrit. The patients are initially place onto Bivalirudin for continuous anticoagulation and may be transitioned to Warfarin and acetylsalicylic acid on a case by case basis.

### Clinical data collection

Clinical data was extracted from the electronic medical record including demographic information, indication for MCS, method of MCS and outcomes. Additionally, we identified the location and causal organism for each new infection. Patients were defined as having a new infection if they developed a new positive blood, urine or respiratory culture for either bacteria or fungi while receiving MCS. All patients classified as developing a new infection had both: 1) new positive culture results and 2) antiobiotic therapy by the clinical team indicating a clinically significant infection. At our institution the clinical practice is to obtain routine blood cultures Monday, Wednesday and Friday while receiving MCS.

### Plasma sample collection and processing

Plasma samples were collected prospectively in EDTA tubes, centrifuged within 30 min at 2500×*g* at 4 °C for 15 min to make platelet poor plasma, aliquoted into 200 microliter samples and frozen at −80 °C until analysis. This included a pre-MCS sample, samples from each day of MCS for the first 7 days and weekly samples after the first week of MCS. Samples were typically obtained in the morning (between 8 and 10 AM), in conjunction with clinically scheduled lab draws. The biomarkers sTREM-1, Presepsin, CRP and Procalcitonin were analyzed utilizing commercially available kits. Plasma concentrations of sTREM-1, Presepsin and Procalcitonin were determined in duplicate for each sample using R&D systems human Trem-1 ELISA kit (Catalog number: DTRM10C), LSBio human Presepsin ELISA kit (Catalog number: LS-F55886) and Abcam human Procalcitonin ELISA kit (Catalog number: ab100630), respectively. Plasma samples which yielded a reading over the calibrator range were re-assayed under appropriate dilution. The reliability of duplicate ELISA analysis was evaluated by calculating the coefficients of variation (CV) for each test point. CV values were categorized as follows: <10% (excellent), 10–15% (acceptable), and >15% (outliers). No outliers (CV > 15%) were identified for sTREM-1 or Presepsin, while one Procalcitonin sample exceeded this threshold. CVs were calculated using Microsoft Excel. CRP was quantified by chemiluminescence using the Immulite 1000 automated chemiluminometer (Siemens Healthcare Diagnostics, Deerfield, IL) with the high-sensitivity CRP kit (Siemens, LKCR1). All concentrations were calculated using GraphPad 9.0 based on four parameter logistic curve fit.

### Statistical analysis

Data is summarized as mean (standard deviation) or median (inter-quartile range and/or range) for continuous variables and frequency (percentage) for categorical variables. Graphical displays of biomarker values over the monitoring timeframe for each biomarker are included. Biomarker values are additionally summarized and presented by MCS modality.

### Analysis of biomarker kinetics

The difference between pre and immediately post cannulation values (day 1 of MCS support only) for each biomarker were compared using Wilcoxon rank sum test, while differences between MCS types were compared using Kruskal-Wallis rank sum test. The differences in each biomarker between pre-MCS and uninfected post MCS initiation time points (all days from: day 1 of MCS support to 48 h prior to the diagnosed infection) were tested using linear mixed-effects models for each biomarker.

### Analysis of biomarker response to infection

The differences in each biomarker between infected and uninfected time points was tested with linear mixed-effects models. For this analysis we utilized each patient as their own control, with uninfected time points for each patient defined as samples >48 h in advance of the diagnosed infection while on MCS, and compared biomarker values to the infected time period [21]. The infected time period for each patient was defined as (the 48 h prior to the time the new positive culture was obtained). This time period, 48 h, was chosen as this is the time in which between 93 and 99% of cultures will be positive if they will grow a pathogenic organism [22–25]. This time period also represents the potential delay of knowing when a patient has a new infection based on the current gold standard diagnostic mechanism of infection: cultures. Clinically, if a culture is negative at 48 h we would be likely to discontinue antimicrobial therapy, thus this time is the period in which the identification of a biomarker that can predict infection reliably on MCS has the most potential benefit. The linear mixed effects models include random intercepts to account for repeated measures within each patient. Youdens J statistic was used to determine estimated cut points for each biomarker, in an exploratory fashion, with the cut points unadjusted for other covariates [26]. One patient was found to have much higher presepsin and procalcitonin values compared to other included patients, however met inclusion criteria and on chart review was not found to be different than the other patients in the cohort. A sensitivity analysis was performed, and their removal from the model did not alter results, therefore data points from this patient were included in the overall analysis.

Hypothesis testing was conducted at a 5% type I error rate (alpha = 0.05). All analyses were conducted in R (Version 4) and all plots were made with ggplot2 [21, 27, 28]. Some patient time points are missing biomarker values due to the timing of infection, absence of sufficient residual sample to run all four biomarkers, or insufficient sample volumes to repeat analysis at appropriate dilutions. Time points that are missing samples are clearly identified within the Supplemental Tables 1–4.

## Results

The existing biobank of 81 patients contained 20 patients who developed a new infection while on MCS. Two patients did not have sufficient residual samples for analysis, leaving a total of 18 patients in the final cohort. The demographics of these 18 patients and details about their acquired infections on MCS are presented in Table 1. There were 9 female patients (50%), most patients were in the cardiac intensive care unit (11/18, 61%), with 1 patient (6%) in the neonatal intensive care unit and 6 patients (33%) in the pediatric intensive care unit, with 3 patients (17%) transitioned to MCS following cardiac surgery due to an inability to separate from cardiopulmonary bypass. The majority of patients were able to be separated from MCS for more than 24 h (11/18, 61%), however 12/18 (67%) of patients did not survive until hospital discharge.

**Table 1:** Patient and infection demographics.

Age	Indication for MCS	Support type	Infection location(s)	Organism(s)	Day of infection	Total days of MCS
7 Years	Lung Transplant Graft Failure	VV	Blood	Enterococcus faecalis and Coagulase Negative Staphylococcus	7	27
58 Days	Post Cardiac Surgery and Failure to Come Off Bypass	VA	Blood/Respiratory	Coagulase Negative Staphylococcus/Klebsiella pneumoniae	8	11
276 Days	Cardiomyopathy/Myocarditis	VA	Blood/Respiratory	Candida albicans/Escheridia coli and Staphylococcus aureus	24	53
159 Days	Pulmonary Hypertension	VV	Respiratory	Staphylococcus aureus	10	14
15 Years	Sepsis	VA	Blood/Respiratory	Candida albicans in both	5	12
63 Days	Sepsis	VA	Respiratory	Stenotrophomonas	5	372
112 Days	Viral Pneumonia	VV	Respiratory	Stenotrophomonas	14	21
13 Years	Cardiomyopathy/Myocarditis	VAD	Respiratory	Staphylococcus aureus	10	34
148 Days	ECPR	VA	Respiratory	Candida species*	4	6
227 Days	Post Cardiac Surgery and Failure to Come Off Bypass	VA	Blood/Respiratory	Pseudomonas/Klebsiella and Serratia	9	9
835 Days	Cardiomyopathy/Myocarditis	VAD	Blood	Streptococcus viridans and Serratia	10	38
126 Days	Combined Bacterial and Viral Pneumonia	VV	Respiratory	Pseudomonas	11	13
1 Day	CDH/PPHN	VA	Blood/Urine	Escherichia coli in both	6	14
240 Days	Pulmonary Hypertension	VAD	Respiratory	Enterobacter cloacae	4	25
6 Years	Hypothermia and Near Drowning	VA	Respiratory	Streptococcus pneumoniae	2	9
25 Years	Cystic Fibrosis Exacerbation	VV	Respiratory	Gram Negative Rods	3	7
90 Days	ECPR	VA	Respiratory	Serratia	5	148
5 Days	Post Cardiac Surgery and Failure to Come Off Bypass	VA	Blood	Staphylococcus hemolyticus	2	3

Legend: Mechanical Circulatory Support (MCS), Veno-arterial ECMO (VA), Ventricular Assist Device (VAD), Veno-veno ECMO (VV), Cannulation to MCS during ongoing cardiopulmonary resuscitation (ECPR), Congenital Diaphragmatic Hernia (CDH), Persistent Pulmonary Hypertension of the Newborn (PPHN). Only patients specified as having been post cardiac surgery and failure to come off bypass were exposed to cardiopulmonary bypass prior to their MCS support. Day of infection represents the number of days from cannulation for mechanical circulatory support, the day before cannulation is day 0, while the first day of mechanical circulatory support accounts for day 1. *Did not speciate to specific candida organism.

Biomarker values pre and post cannulation are presented in Table 2, with no differences identified before and after the cannulation event. We did not identify a difference in pre-cannulation levels for any of the four biomarkers based on mode of support (Supplemental Table 5).

**Table 2:** Pre and post MCS cannulation biomarker kinetics.

Biomarker	Pre-cannulation, *N* = 14^1^	Post-cannulation, *N* = 14^1^	*P*-value^2^
Trem 1 (ng/L)	446 (122, 245, 549, 1354) [488, 339]	371 (183, 264, 463, 898) [390, 185]	0.6
Presepsin (pg/mL)	39,630 (4477, 25,142, 73,225, 318,592) [73,539, 87,577]	46,498 (6267, 24,368, 142,630, 1,059,987) [157,420, 285,461]	0.5
CRP (mg/mL)	31 (1, 1, 112, 676) [111, 196]	61 (19, 44, 187, 632) [131, 167]	0.2
PCT (ng/mL)	0 (0, 0, 1, 94) [7, 25]	1 (0, 0, 2, 57) [5, 15]	0.4

Legend: ^1^Median (Minimum, Interquartile Range, Maximum) [Mean, Standard Deviation], ^2^Wilcoxon rank sum exact test; Wilcoxon rank sum test. Soluble Triggering Receptor expressed on Myeloid cells (sTREM-1), C-Reactive Protein (CRP). *N* is 14 as 4 patients either developed an infection in the first 48 h of mechanical circulatory support or did not have a pre and post cannulation residual sample available for analysis. Due to the use of residual samples, there were some instances of patients missing samples.

Pre-cannulation values of each biomarker were compared to average post-cannulation uninfected values, using liner mixed effects modeling. On average post-cannulation CRP was 542% higher (14.9–95.5 mg/mL) (*P* < 0.001). There was no difference between pre-cannulation and average post-cannulation uninfected values of presepsin, procalcitonin or sTREM-1.

**Figure 1:**
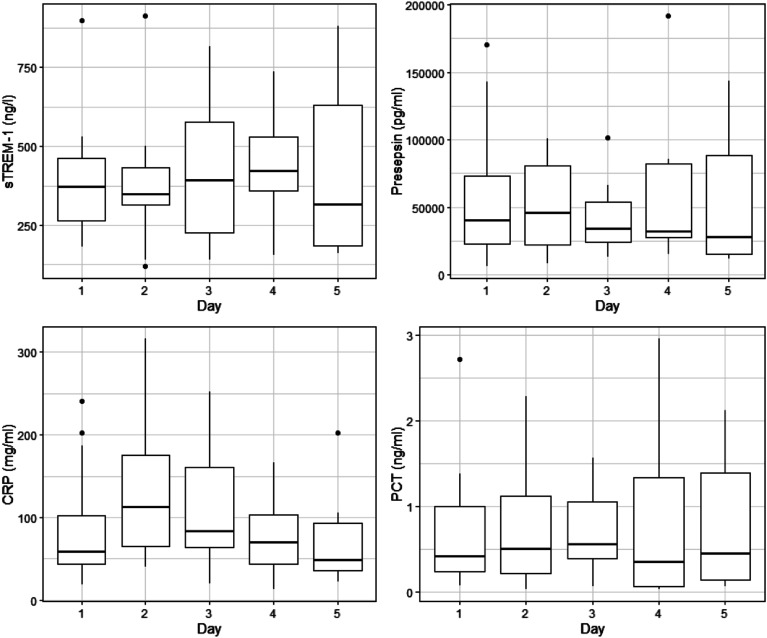
Kinetics of biomarkers while on mechanical circulatory support. Legend: Kinetics of each biomarker during uninfected time periods for the first five days of mechanical circulatory support (Lines indicate median values; boxes indicate 25–75th percentiles; and whiskers indicate the range). Soluble Triggering Receptor expressed on Myeloid cells (sTREM-1), C-Reactive Protein (CRP), Procalcitonin (PCT).

Fourteen patients had samples available during the uninfected time period for examination of biomarker kinetics. The kinetics for the first five days of cannulation are presented in Figure 1 and Supplemental Figure 1. The absolute values for each biomarker over the first five days of support are presented in Table 3. The kinetics of and absolute values for each biomarker based on mode of support for the first five days of cannulation are presented in Supplemental Figure 2 and Supplemental Table 6 respectively.

**Table 3:** Biomarker values while on mechanical circulatory support.

Biomarker	Day 1	Day 2	Day 3	Day 4	Day 5

*N* = 14^1^	*N* = 13^1^	*N* = 10^1^	*N* = 9^1^	*N* = 9^2^
sTREM-1 (ng/L)	371 (183, 264, 463, 898) [390, 185]	367 (120, 325, 498, 1054) [434, 270]	392 (141, 227, 576, 815) [427, 243]	421 (154, 359, 531, 738) [435, 190]	314 (160, 185, 629 880) [435, 277]
Presepsin (pg/mL)	46,498 (6267, 24,368, 142,630, 1,059,987) [157,420, 285,461]	62,389 (8476, 26,010, 100,297, 1,059,987) [155,675, 294,245]	39,496 (12,916, 24,411, 66,552, 396,664) [82,683, 120,826]	31,986 (15,155, 27,876, 82,103, 191,587) [61,354, 58,766]	27,453 (11,537, 15,197, 88,020, 143,358) [55,680, 54,380]
CRP (mg/mL)	61 (19, 44, 187, 632) [131, 167]	112 (41, 80, 316, 544) [196, 168]	119 (21, 77, 236, 556) [194, 191]	90 (13, 45, 166, 544) [171, 191]	80 (23, 40, 288, 1016) [252, 354]
PCT (ng/mL)	1 (0, 0, 2, 57) [5, 15]	1 (0, 0, 2, 189) [16, 52]	0.78 (0.05, 0.42, 1.31, 5.26) [1.19, 1.52]	0.34 (0.03, 0.06, 1.34, 2.96) [0.84, 1.02]	0.44 (0.06, 0.14, 1.39, 2.12) [0.79, 0.82]

Legend: ^1^Median (Minimum, Interquartile Range, Maximum) [Mean, Standard Deviation]. Biomarker values presented represent uninfected time periods for the first five days of mechanical circulatory support. N decreases over time as patients began to develop infections. Soluble Triggering Receptor expressed on myeloid cells (sTREM-1), C-Reactive Protein (CRP). Due to the use of residual samples, there were some instances of patients missing samples.

**Figure 2:**
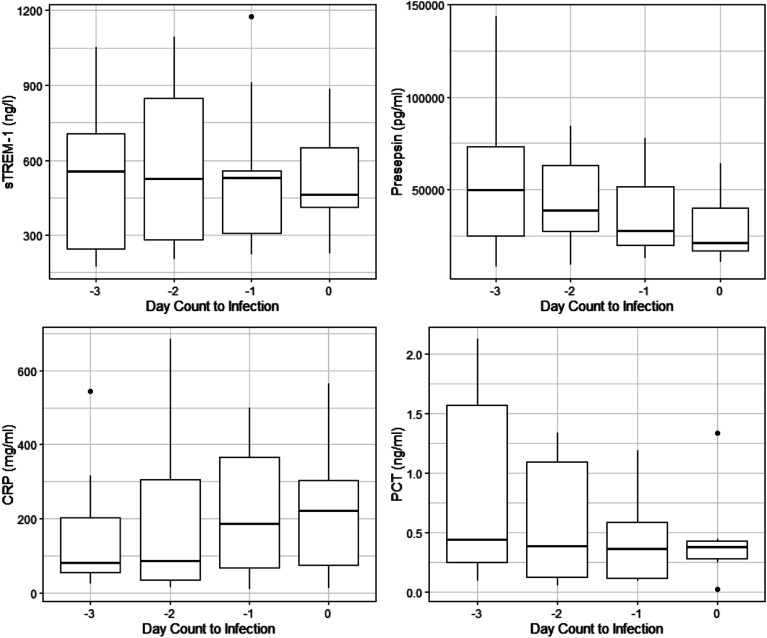
Biomarker response to infection. Legend: Biomarker response to the development of infection (Lines indicate median values; boxes indicate 25–75th percentiles; and whiskers indicate the range). Day-3 represents 72 h in advance of infection (*N* = 10), day-2 represents 48 h in advance of infection (*N* = 10), day-1 represents 24 h in advance of infection (*N* = 10) and day 0 represents the day the culture was obtained that developed an infection (*N* = 7). Soluble Triggering Receptor expressed on Myeloid cells (sTREM-1), C-Reactive Protein (CRP), Procalcitonin (PCT).

Thirteen patients were available for the analysis of the biomarker response to infection on MCS for each of the four biomarkers, displayed in Figure 2 and Supplemental Figure 3. The absolute values of each biomarker in advance of infection are presented in Table 4. Using linear mixed effects modeling we compared the average values for each of the four biomarkers during their post cannulation uninfected periods to the average values during infected periods, ie. the 48 h before infection. On average during periods of infection, Presepsin was 41% lower (51,462–30,188 pg/mL) (*P* = 0.001) and procalcitonin was 51% lower (0.77–0.38 ng/mL) (*P* < 0.001) while there was no difference in sTrem-1 or CRP compared to non-infected periods. To further examine the response of each biomarker to the development of new infection we compared the average biomarker values during the uninfected period (>48 h in advance of the infection) to the biomarker values on the day the positive culture was obtained. In this analysis, only Procalcitonin reached significance and was 37% lower (0.98–0.62 ng/mL) (*P* = 0.04). There was no difference found in this analysis for sTREM-1, CRP or Presepsin.

**Table 4:** Biomarker response to infection.

Biomarker	72 h before infection (Day-3)	48 h before infection (Day-2)	24 h before infection (Day-1)	Day of infection (Day 0)

*N* = 10^1^	*N* = 10^1^	*N* = 10^1^	*N* = 7^1^
sTREM-1 (ng/L)	555 (174, 243, 705, 1054) [531, 305]	524 (205, 283, 849, 1092) [576, 332]	527 (222, 309, 557, 1174) [540, 299]	461 (228, 413, 652, 883) [529, 231]
Presepsin (pg/mL)	58,166 (8476, 26,168, 117,452, 1,059,987) [161,290, 319,011]	49,313 (9627, 27,531, 72,484, 615,634) [102,795, 181,808]	32,334 (13,051, 21,606, 57,477, 524,715) [85,239, 155,820]	24,080 (11,008, 17,324, 54,786, 1,004,613) [169,132, 368,893]
CRP (mg/mL)	131 (25, 56, 288, 1016) [251, 314]	85 (15, 35, 305, 684) [210, 244]	186 (9, 68, 367, 500) [219, 189]	220 (12, 75, 304, 564) [222, 193]
PCT (ng/mL)	1 (0, 0, 2, 189) [20, 59]	0 (0, 0, 1, 131) [14, 41]	0 (0, 0, 1, 72) [8, 23]	0 (0, 0, 1, 41) [6, 15]

Legend: ^1^Median (Minimum, Interquartile Range, Maximum) [Mean, Standard Deviation]. Three patients did not have samples available for analysis on the day of infection due to the use of residual samples. 1 CRP value missing from Day-2 and Day-1. Day of infection represents the day the culture was obtained that developed an infection. Soluble Triggering Receptor expressed on Myeloid cells (sTREM-1), C-Reactive Protein (CRP).

In an exploratory fashion we utilized ROC analysis to determine the optimal cut-point for each of the four biomarkers. The optimal cut-point for sTREM-1 was determined to be 431 ng/L, with an area under the receiver operating characteristic curve (AUC) of 0.56, with a sensitivity of 0.6 and a specificity of 0.54. The optimal cut-point for Presepsin was 64,195 pg/mL with an AUC of 0.553, with a sensitivity of 0.8 and a specificity of 0.36. The optimal cut-point for CRP was 220 mg/mL with an AUC of 0.522, with a sensitivity of 0.39 and a specificity of 0.78. The optimal cut-point for procalcitonin was 0.478 ng/mL with an AUC of 0.618, with a sensitivity of 0.67 and a specificity of 0.59.

## Discussion

To our knowledge this is the first study examining these novel biomarkers, Presepsin and sTREM-1 in MCS patients. We compared and evaluated these novel biomarkers for infection against current commonly used biomarkers CRP and Procalcitonin. We found that levels of these biomarkers were elevated in our patient population, that Presepsin and sTREM-1 did not increase during the cannulation to MCS event and that Presepsin, but not sTREM-1, appeared to have an associated decrease in response to a new infection on MCS.

The patient population of our study is representative overall of children receiving MCS, with a bias towards the Cardiac ICU, consistent with other pediatric literature [1]. The rate of infection within our biobank was 25% (20/81) which was comparable to previously reported infectious complications, ranging from 4.6% in the neonatal MCS population to 42% in the pediatric MCS population [4]. Additionally, our identified patient cohort had a high mortality rate (67%) which is consistent with previous literature demonstrating a high mortality rate in MCS patients who develop infectious complications [2–4].

Interestingly, the pre-cannulation values of sTREM-1 (446 ng/L in our population compared to 105 ng/L) and Presepsin (38,630 pg/mL in our population compared to 3394 pg/mL) were higher than has been previously reported in the literature, albeit our population is slightly older than patients included in previous studies of these biomarkers which focused on primarily septic neonates, and our patients were about to be cannulated to MCS [6, 15, 18, 19, 29]. We attribute these higher levels of novel biomarkers to the illness severity faced by patients who are about to be cannulated for MCS, however there may be additional, not yet understood differences with regards to these biomarkers and how they respond in neonates compared to our slightly older population. It is also possible that given the limitations of our study there is another variable contributing to these difference that we were not able to identify. In two studies examining sTREM-1 and Presepsin in adult patients who received cardiopulmonary bypass for surgery, the values of sTREM-1 in our study were quite similar, while the values of Presepsin were much higher in our patients [13, 30]. We were unable to perform subgroup analysis for neonates as there was only one included in our study.

The immediate post-cannulation values of presepsin, CRP and Procalcitonin were all elevated compared to their pre-cannulation values, while sTREM-1 was lower post-cannulation, however none of these changes reached significance. These increases in CRP and procalcitonin are consistent with previous literature and may be related to the cytokine release that occurs following cannulation [12, 31]. Some literature exists suggesting that MCS itself may impair the innate immune system, of which both sTREM-1 and presepsin are components, and may predispose patients to the development of nosocomial infections [12, 32–34]. While this may explain lower sTREM-1 values it does not provide a clear explanation for the elevated Presepsin values. The average CRP value was significantly greater post cannulation compared to pre cannulation suggesting that MCS itself may increase CRP, making it less useful for the detection of new infection on MCS. Comparatively, average post cannulation procalcitonin was not significantly different from pre cannulation values suggesting that procalcitonin may have utility for the detection of new infection on MCS.

Novel biomarkers sTREM-1 and Presepsin were chosen for this analysis as prior literature has suggested that these may be more specific for new infection rather than an inflammatory response, which we believed would reduce the impact MCS would have on their behavior [6]. In our small pilot study neither sTREM-1 nor Presepsin appear to be impacted by the cannulation event with no difference between the immediate pre and post cannulation values of these two biomarkers. Additionally, neither sTREM-1 nor Presepsin appear to increase or decrease over the period of MCS support and there is no difference between pre cannulation and average post cannulation values for these two biomarkers during uninfected time periods. These data suggest that these biomarkers may be unaffected by the inflammatory response of cannulation for MCS. Our data appears to show a significant decrease in presepsin and procalcitonin in advance of the development of new infection on MCS. This was surprising as this direction of change was unexpected, with published literature describing each of these biomarkers increasing with the development of new infection [6, 9].

After cannulation it appears that there is an initial increase in CRP with subsequent stabilization and relatively unchanged levels of procalcitonin, sTREM-1 and Presepsin. The changes in CRP observed may be explained by the initial inflammatory response to the exposure to foreign substances in the form of the MCS circuit with subsequent normalization of the inflammatory levels consistent with previously published literature [4, 7, 11, 12].

Discovery of biomarkers able to differentiate true infection in the pediatric MCS population is imperative not only to identify and treat true infection more rapidly but also to decrease exposure to antimicrobials in patients who do not need them. Unnecessary use of antimicrobials could result in the development of resistant organisms, making treatment of future infections more challenging. Thus, identifying more reliable biomarkers of infection for patients supported with MCS has the potential to not only improve outcomes but also decrease unnecessary antimicrobial use.

This study is limited in that it is a small pilot study with built in restrictions on sample size and thus power. The sample size is limited as this is a retrospective use of a prospectively collected blood bank with limitations on how many patients developed an infection during their MCS course. The retrospective nature of our study does introduce a selection bias that we were not able to mitigate. A further limitation of our study is that while we utilized each patient as their own uninfected control, we did not have a matched control group who never developed an infection. The absence of this form of control group may lead to unmeasured confounding and other forms of bias. The sum of these limitations require that we clearly state that a larger, more strongly powered study is required before the conclusions and associations between novel biomarkers and infection we have found in our study can or should be utilized clinically.

While false positives, colonization or contamination are possible we believe that our approach of using clinically obtained cultures coupled with the fact that all patients included in our study received antimicrobial therapy as determined by their clinical team, gave us the most accurate definition of infection for this retrospective cohort study, but it is not without some limitations.

The emerging biomarkers sTREM-1 and Presepsin may not be affected by MCS initiation but have significantly higher values than reported in other patient populations. Within our patient population Presepsin and Procalcitonin may have utility in identifying infection but with a decrease rather than the expected increase in their values. These findings highlight the importance of biomarker studies specifically performed in the MCS patient population, and the potential lack of translatability of biomarkers in other patient populations to the MCS patient population.

## Data Availability

All available data is incorporated into the manuscript and the included Supplementary materials.

## Supplementary materials

The supplementary materials of this article are available at https://ject.edpsciences.org/10.1051/ject/2025008/olm. *Supplemental Figure 1*: Kinetics of biomarkers while on mechanical circulatory support. *Supplemental Figure 2*: Kinetics of biomarkers while cannulated by mode of support. *Supplemental Figure 3*: Biomarker response to infection. *Supplemental Table 1*: Pre and post MCS cannulation biomarker kinetics with missing values identified. *Supplemental Table 2*: Pre cannulation levels of biomarkers based on mode of mechanical circulatory support with missing values identified. *Supplemental Table 3*: Biomarker values while on mechanical circulatory support with missing values identified. *Supplemental Table 4*: Biomarker values while cannulated by mode of support with missing values identified. *Supplemental Table 5*: Pre cannulation levels of biomarkers based on mode of mechanical circulatory support. *Supplemental Table 6*: Biomarker values while cannulated by mode of support.
