# Supplementary material for: The utility of sTREM-1 and presepsin to predict infection in pediatric patients receiving mechanical circulatory support
Source: J Extra Corpor Technol. 2025 Jun 16;57(2):96–104. doi: 10.1051/ject/2025008 (PMC12169701; doi:10.1051/ject/2025008)
Supplement: Supplementary file 1 — Supplemental Figure 1: Kinetics of biomarkers while on mechanical circulatory support. Supplemental Figure 2: Kinetics of biomarkers while cannulated by mode of support. Supplemental Figure 3: Biomarker response to infection. Supplemental Table 1: Pre and post MCS cannulation biomarker kinetics with missing values identified. Supplemental Table 2: Pre cannulation levels of biomarkers based on mode of mechanical circulatory support with missing values identified. Supplemental Table 3: Biomarker values while on mechanical circulatory support with missing values identified. Supplemental Table 4: Biomarker values while cannulated by mode of support with missing values identified. Supplemental Table 5: Pre cannulation levels of biomarkers based on mode of mechanical circulatory support. Supplemental Table 6: Biomarker values while cannulated by mode of support. [file ject-57-96-s1.pdf]

Supplemental Tables and Figures

Supplemental Table 1: Pre and Post MCS cannulation Biomarker Kinetics with Missing Values Identified.

**Biomarker**	**Pre-Cannulation**, N = 14[1]	**Post-Cannulation**, N = 14[1]	**p-value**[2]
Trem 1 (ng/l)	446 (122, 245, 549, 1,354) [488, 339]	371 (183, 264, 463, 898) [390, 185]	0.6
Presepsin (pg/ml)	39,630 (4,477, 25,142, 73,225, 318,592) [73,539, 87,577]	46,498 (6,267, 24,368, 142,630, 1,059,987) [157,420, 285,461]	0.5
(Missing)	1	1	
CRP (mg/ml)	31 (1, 1, 112, 676) [111, 196]	61 (19, 44, 187, 632) [131, 167]	0.2
(Missing)	2	1	
PCT (ng/ml)	0 (0, 0, 1, 94) [7, 25]	1 (0, 0, 2, 57) [5, 15]	0.4

Legend: [1]Median (Minimum, Interquartile Range, Maximum) [Mean, Standard Deviation], [2]Wilcoxon rank sum exact test; Wilcoxon rank sum test.. Soluble Triggering Receptor expressed on Myeloid cells (sTREM-1), C-Reactive Protein (CRP). N is 14 as 4 patients either developed an infection in the first 48 hours of mechanical circulatory support or did not have a pre and post cannulation residual sample available for analysis. Due to the use of residual samples, some patients are missing samples, as noted above in this table.

Supplemental Table 2: Pre cannulation levels of biomarkers based on mode of Mechanical Circulatory Support with Missing Values Identified.

**Biomarker**	**VA**, N = 10[1]	**VAD**, N = 3[1]	**VV**, N = 5[1]	**p-value**[2]
Trem 1 (ng/l)	430 (122,311,568, 921) [467, 253]	161 (148,154,311, 462) [257, 178]	463 (223,409,1,354, 2,313) [953, 877]	0.2
(Missing)	1	0	0	
Presepsin (pg/ml)	44,529 (10,715,15,700,135,054, 1,467,133) [234,549, 472,411]	38,851 (4,477,21,664,56,038, 73,225) [38,851, 48,612]	29,840 (25,142,26,410,163,813, 563,431) [161,727, 232,234]	0.7
(Missing)	1	1	0	
CRP (mg/ml)	29 (1,6,262, 676) [160, 239]	17 (1,9,102, 188) [68, 104]	68 (1,37,173, 430) [142, 195]	0.8
(Missing)	2	0	1	
PCT (ng/ml)	0 (0,0,1, 94) [11, 31]	0 (0,0,1, 1) [0, 1]	1 (0,1,1, 12) [3, 5]	0.4
(Missing)	1	0	0	

Legend: [1]Median (Minimum,25%,75%, Maximum) [Mean, SD]), [2]Kruskal-Wallis rank sum test. Veno-arterial ECMO (VA), Ventricular Assist Device (VAD), Veno-veno ECMO (VV), Soluble Triggering Receptor expressed on Myeloid cells (sTREM-1), C-Reactive Protein (CRP). Due to the use of residual samples, some patients are missing samples, as noted above in this table.

Supplemental Table 3: Biomarker Values while on Mechanical Circulatory Support with Missing Values Identified.

	**Day 1**	**Day 2**	**Day 3**	**Day 4**	**Day 5**
**Biomarker**	**N = 14**[1]	**N = 13**[1]	**N = 10**[1]	**N = 9**[1]	**N = 9**[1]
sTREM-1 (ng/L)	371 (183, 264, 463, 898) [390, 185]	367 (120, 325, 498, 1,054) [434, 270]	392 (141, 227, 576, 815) [427, 243]	421 (154, 359, 531, 738) [435, 190]	314 (160, 185, 629 880) [435, 277]
Presepsin (pg/mL)	46,498 (6,267, 24,368, 142,630, 1,059,987) [157,420, 285,461]	62,389 (8,476, 26,010, 100,297, 1,059,987) [155,675, 294,245]	39,496 (12,916, 24,411, 66,552, 396,664) [82,683, 120,826]	31,986 (15,155, 27,876, 82,103, 191,587) [61,354, 58,766]	27,453 (11,537, 15,197, 88,020 143,358) [55,680, 54,380]
(Missing)	1	1	1	1	1
CRP (mg/mL)	61 (19, 44, 187, 632) [131, 167]	112 (41, 80, 316, 544) [196, 168]	119 (21, 77, 236, 556) [194, 191]	90 (13, 45, 166, 544) [171, 191]	80 (23, 40, 288 1,016) [252, 354]
(Missing)	1				1

	Day 1	Day 2	Day 3	Day 4	Day 5
**Biomarker**	**N = 14**[1]	**N = 13**[1]	**N = 10**[1]	**N = 9**[1]	**N = 9**[1]
PCT (ng/mL)	1 (0, 0, 2, 57) [5, 15]	1 (0, 0, 2, 189) [16, 52]	0.78 (0.05, 0.42, 1.31, 5.26) [1.19, 1.52]	0.34 (0.03, 0.06, 1.34, 2.96) [0.84, 1.02]	0.44 (0.06, 0.14, 1.39 2.12) [0.79, 0.82]

Legend: [1]Median (Minimum, Interquartile Range, Maximum) [Mean, Standard Deviation]. Biomarker values presented represent uninfected time periods for the first five days of mechanical circulatory support, separated by mode of support received. Soluble Triggering Receptor expressed on myeloid cells (sTREM-1), C-Reactive Protein (CRP). N decreases over time as patients began to develop infections. Due to the use of residual samples, some patients are missing samples, as noted above in this table.

Supplemental Table 4: Biomarker Values while Cannulated by mode of Support with Missing Values Identified.

	Day 1			Day 2			Day 3			Day 4			Day 5		
**Biomarker**	**VA**, N = 7[1]	**VAD**, N = 3[1]	**VV**, N = 4[1]	**VA**, N = 7[1]	**VAD**, N = 2[1]	**VV**, N = 4[1]	**VA**, N = 4[1]	**VAD**, N = 2[1]	**VV**, N = 4[1]	**VA**, N = 3[1]	**VAD**, N = 2[1]	**VV**, N = 4[1]	**VA**, N = 3[1]	**VAD**, N = 2[1]	**VV**, N = 4[1]
sTREM-1 (ng/L)	392 (245, 335, 488, 530) [402, 105]	189 (183, 186, 308, 428) [267, 140]	371 (205, 326, 506, 898) [461, 301]	330 (281, 327, 456, 1,054) [461, 271]	309 (120, 214, 403, 498) [309, 268]	368 (140, 310, 506, 913) [447, 329]	322 (214, 253, 435, 605) [366, 174]	495 (175, 335, 655, 815) [495, 453]	448 (141, 339, 563, 780) [454, 263]	400 (359, 379, 510, 621) [460, 141]	358 (184, 271, 445, 531) [358, 246]	462 (154, 354, 563, 738) [454, 241]	237 (174, 206, 433, 629) [347, 247]	384 (160, 272, 497, 609) [384, 318]	523 (185, 282, 768, 880) [528, 331]
Presepsin (pg/mL)	70,032 (6,267, 42,667, 109,705, 1,059,987) [205,862, 378,984]	177,627 (24,368, 100,997, 254,257, 330,887) [177,627, 216,742]	30,889 (17,794, 20,553, 72,881, 170,607) [62,545, 72,713]	74,997 (8,476, 45,825, 91,651, 1,059,987) [202,630, 379,264]	285,026 (285,026, 285,026, 285,026, 285,026) [285,026, NA]	24,104 (16,256, 19,282, 45,986, 100,194) [41,164, 39,649]	53,024 (24,411, 35,725, 75,340, 101,703) [58,041, 33,930]	396,664 (396,664, 396,664, 396,664, 396,664) [396,664, NA]	26,206 (12,916, 21,314, 33,722, 49,989) [28,829, 15,529]	80,998 (31,748, 56,373, 83,208, 85,418) [66,055, 29,792]	191,587 (191,587, 191,587, 191,587, 191,587) [191,587, NA]	26,851 (15,155, 22,391, 29,731, 32,224) [25,270, 7,395]	73,304 (14,851, 44,077, 108,331, 143,358) [77,171, 64,341]	132,168 (132,168, 132,168, 132,168, 132,168) [132,168, NA]	20,098 (11,537, 14,369, 26,168, 30,023) [20,439, 8,508]
(Missing)	0	1	0	0	1	0	0	1	0	0	1	0	0	1	0
CRP (mg/mL)	53 (24, 43, 148, 632) [156, 223]	74 (61, 67, 138, 203) [113, 79]	67 (19, 43, 127, 187) [91, 87]	112 (46, 85, 249, 544) [196, 178]	178 (41, 110, 247, 316) [178, 195]	142 (51, 97, 249, 484) [205, 193]	170 (25, 120, 279, 556) [230, 228]	164 (76, 120, 208, 252) [164, 124]	83 (21, 65, 191, 504) [173, 223]	166 (117, 142, 307, 448) [244, 179]	80 (70, 75, 85, 90) [80, 14]	43 (13, 35, 169, 544) [161, 256]	106 (54, 80, 561, 1,016) [392, 541]	122 (42, 82, 162, 202) [122, 113]	34 (23, 28, 289, 544) [200, 298]
(Missing)	0	0	1										0	0	1
PCT (ng/mL)	1 (0, 1, 2, 57) [9, 21]	0 (0, 0, 2, 4) [2, 2]	0 (0, 0, 1, 4) [1, 2]	1 (0, 0, 2, 189) [28, 71]	3 (0, 1, 4, 5) [3, 3]	0 (0, 0, 1, 2) [1, 1]	1.22 (0.39, 0.89, 1.44, 1.57) [1.10, 0.52]	2.66 (0.05, 1.36, 3.96, 5.26) [2.66, 3.68]	0.54 (0.10, 0.43, 0.67, 1.01) [0.55,	1.34 (0.56, 0.95, 1.61, 1.87) [1.26, 0.66]	1.51 (0.05, 0.78, 2.23, 2.96) [1.51,	0.18 (0.03, 0.05, 0.32, 0.34) [0.18, 0.16]	1.39 (0.30, 0.84, 1.69, 1.98) [1.22,	1.09 (0.06, 0.58, 1.60, 2.12) [1.09,	0.29 (0.10, 0.13, 0.47, 0.55) [0.31, 0.22]

	Day 1			Day 2			Day 3			Day 4			Day 5		
**Biomarker**	**VA**, N = 7[1]	**VAD**, N = 3[1]	**VV**, N = 4[1]	**VA**, N = 7[1]	**VAD**, N = 2[1]	**VV**, N = 4[1]	**VA**, N = 4[1]	**VAD**, N = 2[1]	**VV**, N = 4[1]	**VA**, N = 3[1]	**VAD**, N = 2[1]	**VV**, N = 4[1]	**VA**, N = 3[1]	**VAD**, N = 2[1]	**VV**, N = 4[1]
									0.37]		2.05]		0.86]	1.45]	

Legend: [1]Median (IQR). Biomarker values presented represent uninfected time periods for the first five days of mechanical circulatory support. N decreases over time as patients began to develop infections. Soluble Triggering Receptor expressed on myeloid cells (sTREM-1), C-Reactive Protein (CRP). Due to the use of residual samples, some patients are missing samples, as noted above in this table.

Supplemental Table 5: Pre cannulation levels of biomarkers based on mode of Mechanical Circulatory Support.

**Biomarker**	**VA**, N = 10[1]	**VAD**, N = 3[1]	**VV**, N = 5[1]	**p-value**[2]
Trem 1 (ng/l)	430 (122,311,568, 921) [467, 253]	161 (148,154,311, 462) [257, 178]	463 (223,409,1,354, 2,313) [953, 877]	0.2
Presepsin (pg/ml)	44,529 (10,715,15,700,135,054, 1,467,133) [234,549, 472,411]	38,851 (4,477,21,664,56,038, 73,225) [38,851, 48,612]	29,840 (25,142,26,410,163,813, 563,431) [161,727, 232,234]	0.7
CRP (mg/ml)	29 (1,6,262, 676) [160, 239]	17 (1,9,102, 188) [68, 104]	68 (1,37,173, 430) [142, 195]	0.8
PCT (ng/ml)	0 (0,0,1, 94) [11, 31]	0 (0,0,1, 1) [0, 1]	1 (0,1,1, 12) [3, 5]	0.4

Legend: [1]Median (Minimum,25%,75%, Maximum) [Mean, SD][2]Kruskal-Wallis rank sum test. Veno-arterial ECMO (VA), Ventricular Assist Device (VAD), Veno-veno ECMO (VV), Soluble Triggering Receptor expressed on Myeloid cells (sTREM-1), C-Reactive Protein (CRP). Due to the use of residual samples, there were some instances of patients missing samples.

Supplement Table 6: Biomarker Values while Cannulated by mode of Support.

	Day 1			Day 2			Day 3			Day 4			Day 5		
**Biomarker**	**VA**, N = 7[1]	**VAD**, N = 3[1]	**VV**, N = 4[1]	**VA**, N = 7[1]	**VAD**, N = 2[1]	**VV**, N = 4[1]	**VA**, N = 4[1]	**VAD**, N = 2[1]	**VV**, N = 4[1]	**VA**, N = 3[1]	**VAD**, N = 2[1]	**VV**, N = 4[1]	**VA**, N = 3[1]	**VAD**, N = 2[1]	**VV**, N = 4[1]

	Day 1			Day 2			Day 3			Day 4			Day 5		
**Biomarker**	**VA**, N = 7[1]	**VAD**, N = 3[1]	**VV**, N = 4[1]	**VA**, N = 7[1]	**VAD**, N = 2[1]	**VV**, N = 4[1]	**VA**, N = 4[1]	**VAD**, N = 2[1]	**VV**, N = 4[1]	**VA**, N = 3[1]	**VAD**, N = 2[1]	**VV**, N = 4[1]	**VA**, N = 3[1]	**VAD**, N = 2[1]	**VV**, N = 4[1]
sTREM-1 (ng/L)	392 (245, 335, 488, 530) [402, 105]	189 (183, 186, 308, 428) [267, 140]	371 (205, 326, 506, 898) [461, 301]	330 (281, 327, 456, 1,054) [461, 271]	309 (120, 214, 403, 498) [309, 268]	368 (140, 310, 506, 913) [447, 329]	322 (214, 253, 435, 605) [366, 174]	495 (175, 335, 655, 815) [495, 453]	448 (141, 339, 563, 780) [454, 263]	400 (359, 379, 510, 621) [460, 141]	358 (184, 271, 445, 531) [358, 246]	462 (154, 354, 563, 738) [454, 241]	237 (174, 206, 433, 629) [347, 247]	384 (160, 272, 497, 609) [384, 318]	523 (185, 282, 768, 880) [528, 331]
Presepsin (pg/mL)	70,032 (6,267, 42,667, 109,705, 1,059,987) [205,862, 378,984]	177,627 (24,368, 100,997, 254,257, 330,887) [177,627, 216,742]	30,889 (17,794, 20,553, 72,881, 170,607) [62,545, 72,713]	74,997 (8,476, 45,825, 91,651, 1,059,987) [202,630, 379,264]	285,026 (285,026, 285,026, 285,026, 285,026) [285,026, NA]	24,104 (16,256, 19,282, 45,986, 100,194) [41,164, 39,649]	53,024 (24,411, 35,725, 75,340, 101,703) [58,041, 33,930]	396,664 (396,664, 396,664, 396,664, 396,664) [396,664, NA]	26,206 (12,916, 21,314, 33,722, 49,989) [28,829, 15,529]	80,998 (31,748, 56,373, 83,208, 85,418) [66,055, 29,792]	191,587 (191,587 , 191,587, 191,587, 191,587) [191,587 , NA]	26,851 (15,155, 22,391, 29,731, 32,224) [25,270, 7,395]	73,304 (14,851, 44,077, 108,331, 143,358) [77,171, 64,341]	132,168 (132,168, 132,168, 132,168, 132,168) [132,168, NA]	20,098 (11,537, 14,369, 26,168, 30,023) [20,439, 8,508]
CRP (mg/mL)	53 (24, 43, 148, 632) [156, 223]	74 (61, 67, 138, 203) [113, 79]	67 (19, 43, 127, 187) [91, 87]	112 (46, 85, 249, 544) [196, 178]	178 (41, 110, 247, 316) [178, 195]	142 (51, 97, 249, 484) [205, 193]	170 (25, 120, 279, 556) [230, 228]	164 (76, 120, 208, 252) [164, 124]	83 (21, 65, 191, 504) [173, 223]	166 (117, 142, 307, 448) [244, 179]	80 (70, 75, 85, 90) [80, 14]	43 (13, 35, 169, 544) [161, 256]	106 (54, 80, 561, 1,016) [392, 541]	122 (42, 82, 162, 202) [122, 113]	34 (23, 28, 289, 544) [200, 298]
PCT (ng/mL)	1 (0, 1, 2, 57) [9, 21]	0 (0, 0, 2, 4) [2, 2]	0 (0, 0, 1, 4) [1, 2]	1 (0, 0, 2, 189) [28, 71]	3 (0, 1, 4, 5) [3, 3]	0 (0, 0, 1, 2) [1, 1]	1.22 (0.39, 0.89, 1.44, 1.57) [1.10, 0.52]	2.66 (0.05, 1.36, 3.96, 5.26) [2.66, 3.68]	0.54 (0.10, 0.43, 0.67, 1.01) [0.55, 0.37]	1.34 (0.56, 0.95, 1.61, 1.87) [1.26, 0.66]	1.51 (0.05, 0.78, 2.23, 2.96) [1.51, 2.05]	0.18 (0.03, 0.05, 0.32, 0.34) [0.18, 0.16]	1.39 (0.30, 0.84, 1.69, 1.98) [1.22, 0.86]	1.09 (0.06, 0.58, 1.60, 2.12) [1.09, 1.45]	0.29 (0.10, 0.13, 0.47, 0.55) [0.31, 0.22]

Legend: [1]Median (Range). Biomarker values presented represent uninfected time periods for the first five days of mechanical circulatory support, separated by mode of support received. Soluble Triggering Receptor expressed on myeloid cells (sTREM-1), C-Reactive Protein (CRP). N decreases over time as patients began to develop infections. Due to the use of residual samples, there were some instances of missing samples.

Supplemental Figure 1: Kinetics of Biomarkers while on Mechanical Circulatory Support.

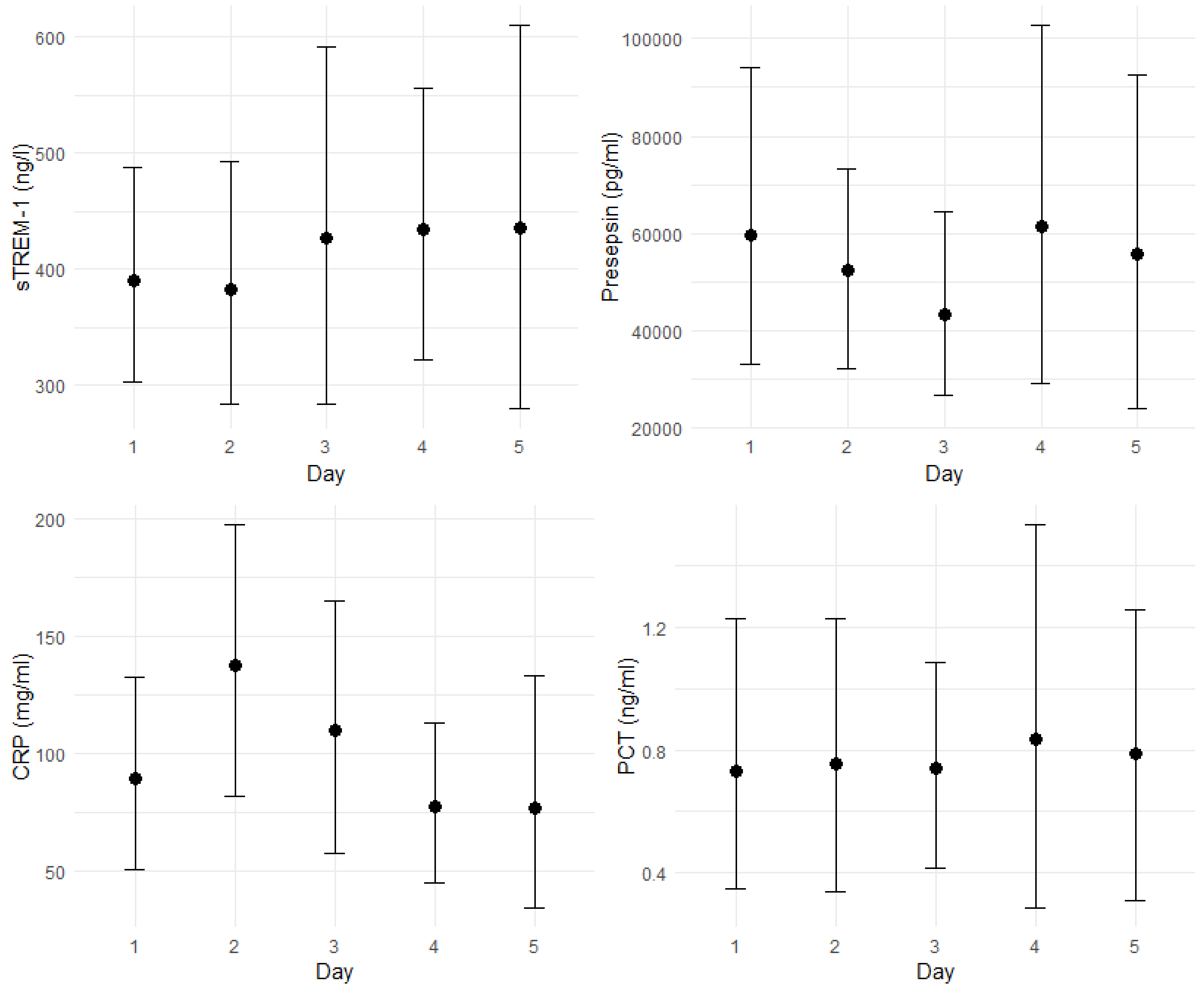

Legend: Mean and 95% Confidence interval. For viewability Y axis does not include 0 for all biomarkers. Soluble Triggering Receptor expressed on Myeloid cells (sTREM-1), C-Reactive Protein (CRP), Procalcitonin (PCT).

Supplemental Figure 2: Kinetics of Biomarkers while cannulated by mode of Support.

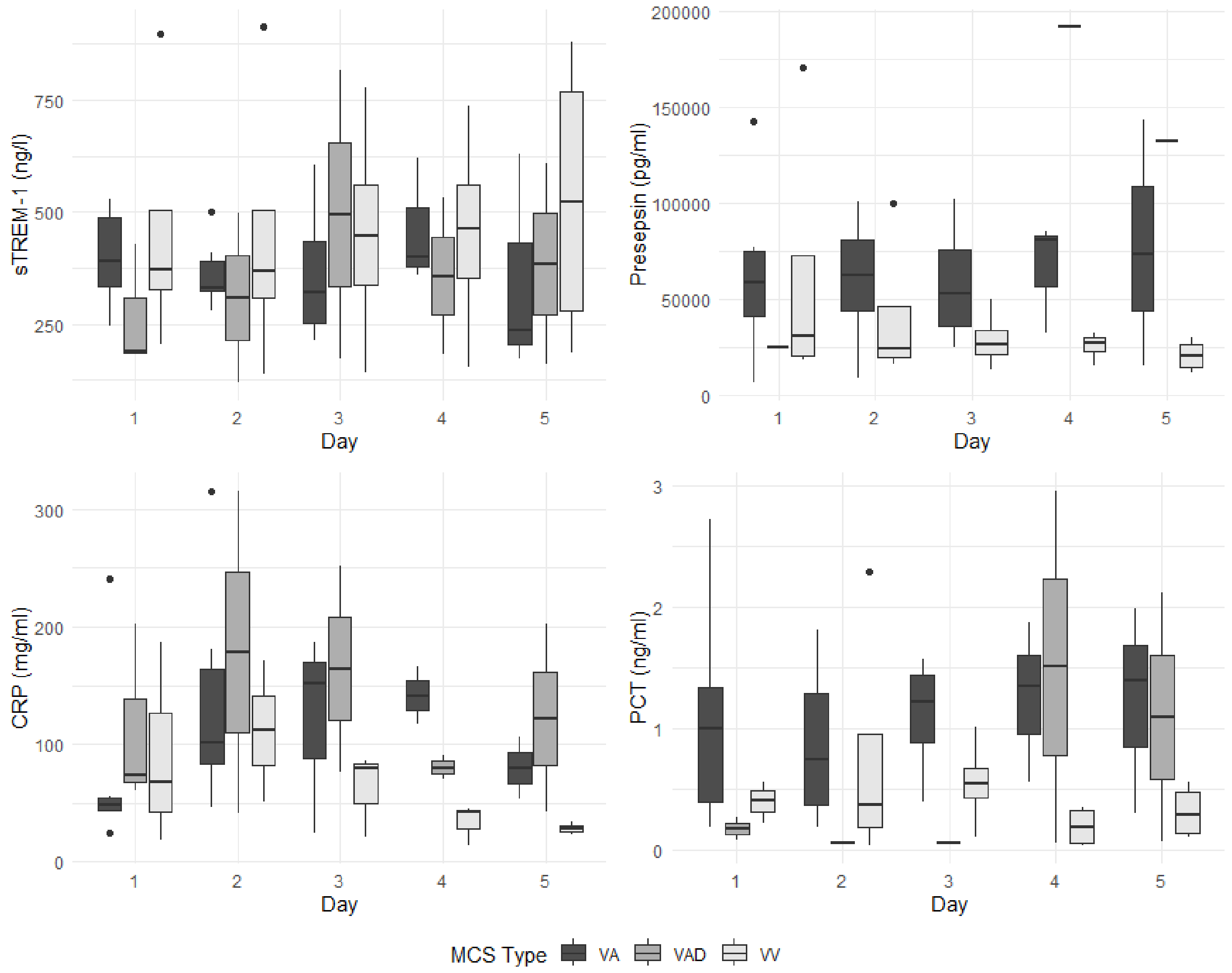

Legend: Kinetics of each biomarker during uninfected time periods during the first five days of mechanical circulatory support, presented by mode of support (Lines indicate median values; boxes indicate 25th-75th percentiles; and whiskers indicate the range). Veno-arterial ECMO (VA), Ventricular Assist Device (VAD), Veno-veno ECMO (VV), Soluble Triggering Receptor expressed on Myeloid cells (sTREM-1), C-Reactive Protein (CRP), Procalcitonin (PCT).

Supplemental Figure 3: Biomarker Response to infection.

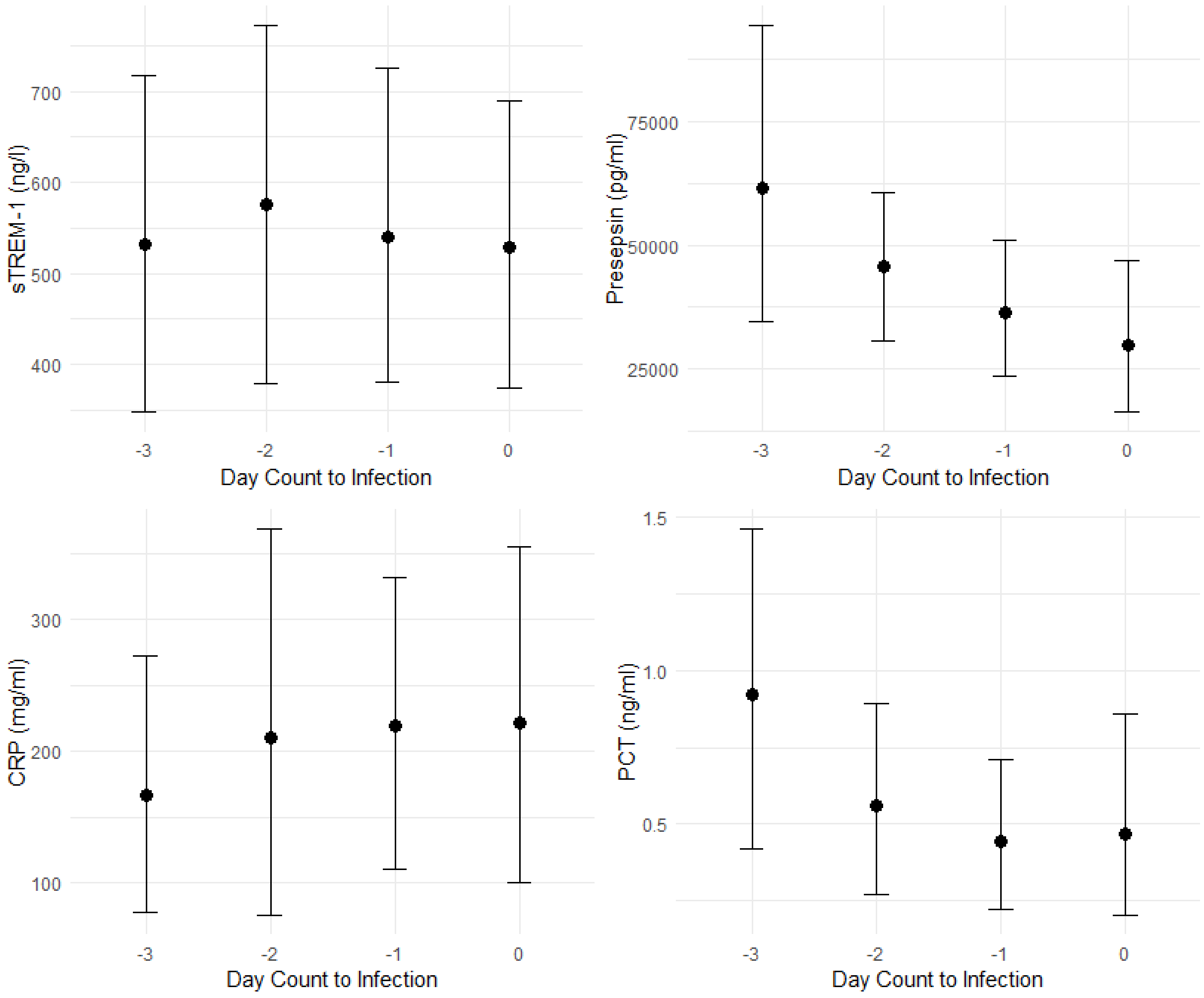

sTREM-1 (ng/l)
700
600
500
400
-3
-2
-1
0
Day Count to Infection
Presepsin (pg/ml)
75000
50000
25000
-3
-2
-1
0
Day Count to Infection
CRP (mg/ml)
300
200
100
-3
-2
-1
0
Day Count to Infection
PCT (ng/ml)
1.5
1.0
0.5
-3
-2
-1
0
Day Count to Infection

Legend: Mean and 95% Confidence interval. For viewability Y axis does not include 0 for all biomarkers. Day -3 represents 72 hours in advance of infection (N=10), day -2 represents 48 hours in advance of infection (N=10), day -1 represents 24 hours in advance of infection (N=10) and day 0 represents the day the culture was obtained that developed an infection (N=7). Soluble Triggering Receptor expressed on Myeloid cells (sTREM-1), C-Reactive Protein (CRP), Procalcitonin (PCT).
